# Simultaneous Determination of Fourteen Marker Compounds in the Traditional Herbal Prescription, Geumgwesingihwan, Using Ultra-Performance Liquid Chromatography–Tandem Mass Spectrometry

**DOI:** 10.3390/molecules27123890

**Published:** 2022-06-17

**Authors:** Chang-Seob Seo, Mee-Young Lee

**Affiliations:** 1 KM Science Research Division, Korea Institute of Oriental Medicine, Daejeon 34054, Korea; 2 KM Convergence Research Division, Korea Institute of Oriental Medicine, Daejeon 34054, Korea; cozy11@kiom.re.kr

**Keywords:** Geumgwesingihwan, traditional herbal prescription, simultaneous determination, UPLC–MS/MS

## Abstract

Geumgwesingihwan (GSH) is a traditional herbal prescription composed of eight medicinal herbs: *Rehmannia glutinosa* (Gaertn.) DC., *Dioscorea japonica* Thunb., *Cornus officinalis* Siebold and Zucc., *Poria cocos* Wolf, *Paeonia suffruticosa* Andrews, *Alisma plantago-aquatica* subsp. *orientale* (Sam.) Sam., *Achyranthes bidentate* Blume, and *Plantago asiatica* L. This study developed and validated an ultra-performance liquid chromatography–tandem mass spectrometry (UPLC–MS/MS) method in the multiple reaction monitoring (MRM) mode for simultaneous determination of 14 compounds (allantoin, gallic acid, 5-(hydroxymethyl)furfural, geniposidic acid, oxypaeoniflorin, loganin, geniposide, paeoniflorin, ecdysterone, verbascoside, cornuside, benzoylpaeoniflorin, paeonol, and alisol B acetate) in GSH. The chromatographic separation of all marker analytes was carried out on an Acquity UPLC BEH C_18_ column (100 mm × 2.1 mm, 1.7 µm) using gradient elution of a mobile phase of distilled water–acetonitrile containing 0.1% acetic acid. The newly established UPLC–MS/MS MRM method was validated by evaluating the linearity, the limits of detection and quantification, recovery, and precision. All markers were detected at concentrations of 6.94–4126.28 mg/kg. In addition, the recovery was 76.65–119.49% and the relative standard deviation value of the precision was 0.19–9.91%. The newly developed and validated UPLC–MS/MS assay will provide useful information for quality assessment of GSH.

## 1. Introduction

Traditional Chinese medicine (TCM), traditional Korean medicine (TKM), and Kampo medicine (KM) prescriptions are a complex composed of two or more herbal medicines. They have been widely manufactured and administered in Asian countries such as China, Korea, and Japan for the treatment of various diseases or health maintenance and promotion because of the multitarget feature by multicomponents [1].

Geumgwesingihwan (GSH) has been used for the treatment of edema [2]. GSH is recorded in “Bangyakhappyeon”, a medical book written by Hwang during the Joseon Dynasty, and consists of eight herbal medicines: *Rehmannia glutinosa* (Gaertn.) DC., *Dioscorea japonica* Thunb., *Cornus officinalis* Siebold and Zucc., *Poria cocos* Wolf, *Paeonia suffruticosa* Andrews, *Alisma plantago-aquatica* subsp. *orientale* (Sam.) Sam., *Achyranthes bidentate* Blume, and *Plantago asiatica* L. [2].

For the quality control of TCMs, TKMs, and KMs composed of several herbal medicines, it is by no means easy to analyze the numerous components contained in them at the same time. Nevertheless, many studies have been conducted to achieve standardization of raw materials using high-performance liquid chromatography (HPLC) coupled with an ultraviolet detector, evaporative light scattering detector, photodiode array detector, gas chromatography–mass spectrometry (GC–MS), and liquid chromatography with tandem mass spectrometry (LC–MS/MS) methods [3,4,5,6,7]. Most of these methods took a long time to analyze, targeted specific components, or focused on qualitative analysis. In addition, simultaneous analysis studies on each herbal medicine constituting GSH were also reported using various analytical equipment such as HPLC and LC–MS [8,9,10,11,12,13,14,15]. However, an assay for quality control of GSH has not been reported.

Therefore, in this study, 14 marker components—allantoin (**1**), gallic acid (**2**), 5-(hydroxymethyl)furfural (**3**), geniposidic acid (**4**), oxypaeoniflorin (**5**), loganin (**6**), geniposide (**7**), paeoniflorin (**8**), ecdysterone (**9**), verbascoside (**10**), cornuside (**11**), benzoylpaeoniflorin (**12**), paeonol (**13**), and alisol B acetate (**14**)—in GSH were analyzed simultaneously using ultra-performance liquid chromatography–tandem mass spectrometry (UPLC–MS/MS).

## 2. Results and Discussion

### 2.1. Selection of Marker Components for Simultaneous Analysis Using UPLC–MS/MS

We performed a profiling analysis using an UPLC–MS/MS multiple reaction monitoring (MRM) method on the following components to determine the marker analytes for quality control of GSH: 5-(hydroxymethyl)furfural from *R. glutinosa*; allantoin and dioscin from *D. japonica*; gallic acid, loganin, morroniside, sweroside, cornin, and cornuside from *C. officinalis*; pachymic acid and polyporenic acid C from *P. cocos*; paeonol, paeoniflorin, benzoic acid, oxypaeoniflorin, and benzoylpaeoniflorin from *P. suffruticosa*; alisol B and alisol B acetate from *A. orientale*; ecdysterone from *A. bidentate*; and geniposide, geniposidic acid, and verbascoside from *P. asiatica* [8,9,10,11,12,13,14,15,16,17,18]. We attempted to detect a total of 21 components selected from each constituent herbal medicine of GSH in the sample. Figure S1 in Supplementary Materials shows the UPLC–MS/MS MRM chromatograms of these marker candidates in the positive and negative ion modes, and only 14 components were detected in the GSH sample. Therefore, we selected these 14 detected components as marker analytes for quality control of GSH.

### 2.2. Optimization of UPLC Operation Conditions and UPLC–MS/MS MRM Parameters for Simultaneous Analysis

**Figure 1:**
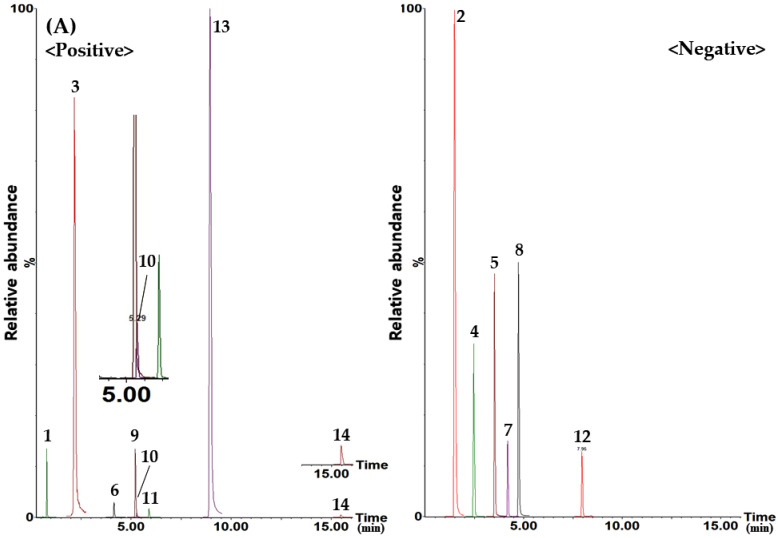

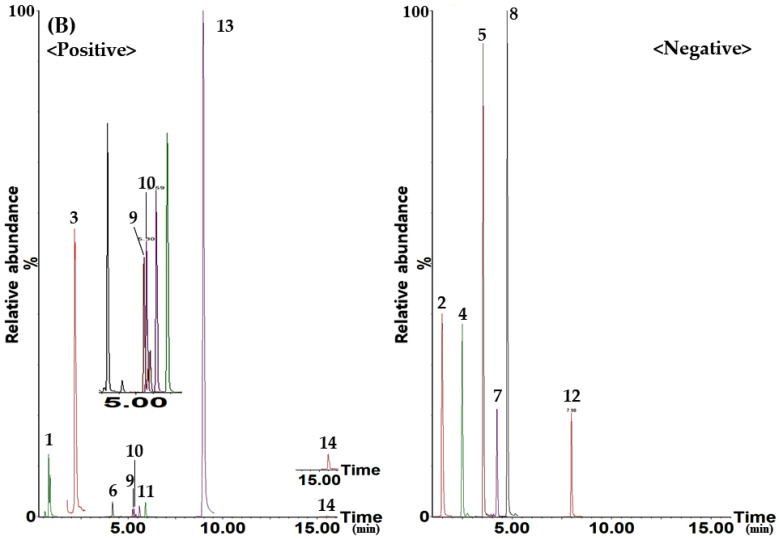
UPLC–MS/MS MRM chromatograms of the standard solution (**A**) and GSH sample (**B**). Allantoin (**1**, 10.77 µg/L), gallic acid (**2**, 200.00 µg/L), 5-(hydroxymethyl)furfural (**3**, 65.56 µg/L), geniposidic acid (**4**, 6.86 µg/L), oxypaeoniflorin (**5**, 7.57 µg/L), loganin (**6**, 22.86 µg/L), geniposide (**7**, 8.00 µg/L), paeoniflorin (**8**, 43.08 µg/L), ecdysterone (**9**, 6.43 µg/L), verbascoside (**10**, 3.83 µg/L), cornuside (**11**, 4.00 µg/L), benzoylpaeoniflorin (**12**, 8.00 µg/L), paeonol (**13**, 30.19 µg/L), and alisol B acetate (**14**, 1.50 µg/L).

Compounds **1**–**14** were isolated and eluted from all markers within 20 min on an Acquity UPLC BEH C_18_ reversed-phase column (2.1 mm × 100 mm, 1.7 µm; Waters, Milford, MA, USA) maintained at 40 °C and gradient elution of a mobile phase system of distilled water–acetonitrile (both containing 0.1% acetic acid) (Table S1). UPLC–MS/MS MRM conditions for simultaneous quantification of 14 marker analytes selected for the quality evaluation of GSH were explored. For quantification of the product ion (Q3) from the precursor ion (Q1) of each marker under the optimized MRM conditions of the markers, the MRM peak data were acquired for approximately 0.5 min at the retention time of each marker, and detailed parameters are presented in Table 1. Figure 1 shows the UPLC–MS/MS MRM chromatograms obtained in the positive and negative ion modes by applying optimized analysis conditions. The blank chromatogram of each analyte is shown in Figure S2.

### 2.3. Identification of Each Marker Analyte for UPLC–MS/MS MRM Quantification

MRM conditions were applied for simultaneous analysis of compounds **1**–**14** in GSH samples using UPLC–MS/MS. Compounds **1**, **3**, **6**, **9**–**11**, **13**, and **14** were detected at 0.79, 2.19, 4.17, 5.25, 5.32, 5.92, 8.98, and 15.53 min at *m/z* 158.9, 126.9, 391.1, 481.2, 625.1, 543.1, 166.9, and 515.3 in the positive ion mode, respectively. The remaining six components, compounds **2**, **4**, **5**, **7**, **8**, and **12**, were detected at 1.47, 2.48, 3.53, 4.20, 4.76, and 7.99 min at *m/z* 169.0, 373.0, 495.0, 446.9, 478.9, and 583.0 in the negative ion mode, respectively. As shown in Table 1, Q1/Q3 peaks were set for each marker analyte for UPLC–MS/MS MRM simultaneous analysis. The Q3 peaks of compounds **1** and **2** were set to *m/z* 115.9 and 125.0 in the form of [M + H − CONH_2_]^+^ and [M − H − COO^−^]^−^ in which CONH_2_ and COO^−^ were lost from the Q1 peaks, respectively [19,20]. The Q3 ion peaks of compounds **3** and **9** were detected at *m/z* 109.0 ([M + H − H_2_O]^+^) and 445.1 ([M + H − 2H_2_O]^+^), respectively [21,22]. The Q3 peak of compound **4** was set at *m/z* 122.9 ([M − H − Glu−H_2_O]^−^) by Retro-Diels–Alder cleavage [23]. The Q3 ion peak of compound **5** was set at *m/z* 136.9 ([M − H − CH_2_O − (*p*-hydroxybenzoic acid) − Glu − CO]^−^) [24]. For compounds **6** and **7** of the iridoid series, Q1 peaks were detected at *m/z* 391.1 and 446.9 in the form of [M + H]^+^ and [M − H + CH_3_OO]^−^, respectively. There was an aglycone in which the glucose group was lost, and the Q3 peaks were detected at *m/z* 228.9 ([M + H − Glu]^+^) and 224.9 ([M − H − Glu]^−^), respectively [23,25]. The Q3 peaks for compounds **8** and **12** were set at *m/z* 448.9 ([M − H − CHOH]^−^) and 553.0 ([M − H − CHOH]^−^) with 30 Da (CHOH) removed, respectively [20]. The Q3 peaks for compounds **10**, **13**, and **14** were detected at *m/z* 163.0, 42.9, and 97.0 from which C_20_H_29_O_12_, C_7_H_7_O_2_, and C_26_H_40_O_4_ were eliminated, respectively [26,27]. In compound **11**, the Q3 peak was detected at *m/z* 211.0 ([M + H − Glu − trihydroxybenzoic acid]^+^) from which glucose and trihydroxybenzoic acid groups were eliminated from the Q1 peak of *m/z* 543.1 in the form of [M + H]^+^ [28]. The Q1/Q3 mass spectra of 14 marker components are presented in Figure S3.

### 2.4. Method Validation of the Developed UPLC–MS/MS MRM Assay

The appropriateness of the UPLC–MS/MS MRM simultaneous analysis method developed for efficient quality control of GSH was validated by evaluating the linearity, limit of detection (LOD), limit of quantification (LOQ), recovery, and precision. In the newly developed analysis method, the linearity was evaluated by the coefficient of determination (*r*^2^) using the calibration curve of each marker analyte, and compounds **1**–**14** showed good linearity, with *r*^2^ ≥ 0.9905 and residuals < 10.0% (Table 2 and Figure S4). In addition, the LOD and LOQ values were calculated as 0.01–5.13 µg/L and 0.02–15.40 µg/L, respectively. Detailed results of each marker analyte are shown in Table 2. The LOD chromatogram is shown in Figure S5. The recoveries (%) of compounds **1**–**14** performed at three different concentrations were measured as 76.65–119.64% (Table 3). Table 4 and Table S2 show the validation values of repeatability and intra- and inter-day precision, which were evaluated using the coefficient of variation (CV, %). The CV values of the repeatability for the retention times of compounds **1**–**14** were 0.03–1.57% (Table S2) and intra- and inter-day precision showed CV values of <10.0% (Table 4). All validation parameters such as the linearity, LOD, LOQ, recovery, and precision showed good results, suggesting that the UPLC–MS/MS analysis method used for the simultaneous analysis of GSH in this study was properly developed.

### 2.5. Quantification of Compounds **1**–**14** in GSH Samples by UPLC–MS/MS MRM Assay

The newly developed UPLC–MS/MS method in this study was successfully applied to the quantitative analysis of compounds **1**–**14** in GSH samples. All marker analytes were completely eluted within 16 min in the positive and negative ion modes of the electrospray ionization source using the highly accurate and sensitive UPLC–MS/MS MRM analytical method (Table 1, Figure 1 and Figure S6). Compounds **1**–**14** were detected at concentrations of 6.94–4126.86 mg/kg, and among them, compounds **1**–**3**, **6**, **8**, and **13** were contained in relatively large amounts in GSH samples compared with other marker analytes (Table 5).

## 3. Materials and Methods

### 3.1. Plant Materials

As shown in Table S3, eight medicinal herbs constituting GSH were purchased from Kwangmyungdang Pharmaceutical (Ulsan, Korea) in November 2017. Each raw medicinal herb was morphologically identified according to the guideline “The Dispensatory on the Visual and Organoleptic Examination of Herbal Medicine” by Dr. Goya Choi, Korea Institute of Oriental Medicine (KIOM, Daejeon, Korea) [29]. Specimens (2018CA04–1 to 2018CA04–8) of each raw material have been deposited at the KM Science Research Division, KIOM.

### 3.2. Chemicals and Reagents

Fourteen reference standard components (compounds **1**–**14**, Figure S7), which are marker analytes used for quality evaluation of GSH samples, were purchased from companies that specialize in standard compounds: compounds **1** (allantoin, 99.7%, Catalog No. 5670), **2** (gallic acid, 100.0%, Catalog No. G7384), **3** (5-(hydroxymethyl)-furfural, ≥99.9%, Catalog No. W501808), and **13** (paeonol, 99.9%, Catalog No. H35803) from Merck KgaA (Darmstadt, Germany); compounds **4** (geniposidic acid, ≥98.0%, Catalog No. 078-05841), **6** (loganin, 98.0%, Catalog No. 125-03621), **7** (geniposide, ≥98.0%, Catalog No. 073-05891), and **14** (alisol B acetate, 99.1%, Catalog No. 018-13231) from Fujifilm Wako Pure Chemical Co., Ltd. (Osaka, Japan); compounds **5** (oxypaeoniflorin, ≥98.0%, Catalog No. DR10581), **8** (paeoniflorin, 99.4%, Catalog No.DR10579), **11** (cornuside, 98.7%, Catalog No.DR10598), and **12** (benzoylpaeoniflorin, ≥98.0%, Catalog No. DR10582) from Shanghai Sunny Biotech Co., Ltd. (Shanghai, China); and compounds **9** (ecdysterone, 98.1%, Catalog No. BP0262) and **10** (verbascoside, 99.7%, Catalog No. BP0124) from Biopurify Phytochemicals (Chengdu, China). HPLC-grade solvents, methanol and acetonitrile, were purchased from J.T. Baker Chemical Co (Phillipsburg, NJ, USA). Water was purified at 18.2 MΩ using an EXL^®^5 ultra water system (Vivagen Co., Ltd., Seongnam, Korea). Glacial acetic acid (≥100.0%, ACS reagent-grade) was purchased from Merck KgaA (Darmstadt, Germany).

### 3.3. Preparation of GSH Water Extract

GSH water extract (GSH–1) was prepared in KIOM according to the sample preparation protocol of a previously reported study [30,31]. Briefly, after mixing the eight herbal medicines based on the weight presented in Table S3, 50 L of water was added and extracted at 100 °C for 2 h. To obtain a powder sample, the extract was freeze-dried (GSH–1: 1355.6 g; yield: 27.1%). The prepared sample was refrigerated until use. Another water extract of GSH (GSH–2) was provided by the College of Oriental Medicine, Wonkwang University.

### 3.4. Preparation of Sample Solutions and Standard Solutions for UPLC–MS/MS MRM Analysis

Sample solutions for UPLC–MS/MS MRM analysis of compounds **1**–**14** in GSH samples were prepared at concentrations of 142.0 mg/100 mL (GSH–1) and 133.0 mg/100 mL (GSH–2) using 50% methanol as solvent. Compound **3** was used for quantitation by diluting the prepared sample solution 10-fold.

Standard solutions of each reference standard compound were prepared at the following concentrations using methanol as the solvent: compounds **1** and **8** (2600 µg/mL), compounds **2** and **3** (3600 µg/mL), compound **4** (3500 µg/mL), compound **5** (3700 µg/mL), compound **6** (2800 µg/mL), compounds **7**, **11**, and **12** (3000 µg/mL), compound **9** (2800 µg/mL), compound **10** (2300 µg/mL), compound **13** (5300 µg/mL), and compound **14** (3200 µg/mL) and diluted before use while refrigerated.

These prepared solutions were filtered through a 0.22 µm membrane filter (Pall Life Sciences, Ann Arbor, MI, USA) before UPLC–MS/MS analysis.

### 3.5. UPLC–MS/MS MRM Analysis Conditions for Simultaneous Determination of the 14 Marker Analytes in GSH

UPLC–MS/MS MRM analysis for quantitative analysis of compounds **1**–**14** was applied to GSH samples by modifying the previously reported analysis protocol [31]. Briefly, a Waters Acquity UPLC H-Class (Milford, MA, USA) coupled with Xevo TQ-S micro MS system (Milford, MA, USA) was used, which was data accumulated and controlled using MassLynx (version 4.2; Milford, MA, USA). Compounds **1**–**14** were separated and quantified using an Acquity UPLC BEH C_18_ column (2.1 mm × 100 mm, 1.7 µm; Milford, MA, USA) and gradient elution of a distilled water–acetonitrile mobile phase system both containing 0.1% (*v*/*v*) acetic acid. Detailed operating parameters of the equipment are shown in Table S1, and the MRM analysis conditions of each marker analyte for simultaneous analysis are summarized in Table 1.

### 3.6. Method Validation of the Established UPLC–MS/MS MRM Assay for Quality Control of GSH

Various factors such as the linearity, LOD, LOQ, recovery, and precision were investigated based on the guidelines to develop a simultaneous UPLC–MS/MS analysis method for compounds **1**–**14** in GSH samples and to validate the analysis method [32]. The linearity was validated by the *r*^2^ value of the calibration curve prepared in the linearity range of each marker analyte shown in Table 2, and ≥0.99 was set as an appropriate range. At the same time, the LOD and LOQ values were calculated using the signal-to-noise ratio (S/N): LOD = 3 × S/N and LOQ = LOD × 3. Next, the recovery was evaluated using the standard addition method. That is, extraction and analysis were conducted by adding three different concentrations (low, medium, and high) of each known marker analyte to the GSH sample, and the recovery was calculated: recovery (%) = (found amount/spiked amount) × 100. The repeatability was measured six times using a standard solution, and then the CV value for the retention time of each marker analyte was evaluated. In addition, intra- and inter-day precisions were assessed using the CV values of compounds **1**–**14** measured for one day and three consecutive days, respectively.

## 4. Conclusions

In the present study, a highly accurate and sensitive UPLC–MS/MS system was firstly developed for simultaneous analysis of compounds **1**–**14** and to use it as basic data for quality control of a traditional herbal prescription, GSH. The newly UPLC–MS/MS MRM analytical method was developed satisfactorily, and the developed assay was validated by examining the linearity, LOD, LOQ, recovery, and precision. Furthermore, this method will be used as basic data for setting up a method for evaluating the quality of other TCMs, TKMs, and KMs prescriptions as well as GSH.

## Figures and Tables

**Table 1:** MRM parameters of each marker analyte for UPLC–MS/MS analysis.

Analyte	Ion Mode	Exact Mass (Da)	Precursor Ion (Q1, *m/z*)	Product Ion (Q3, *m/z*)	Cone Voltage (V)	Collision Energy (eV)	Retention Time (min)	Time Window (min)
**1**	+	158.04	158.9	115.9	14	5	0.79	0.30–1.30
**2**	−	170.02	169.0	125.0	40	13	1.47	1.00–2.00
**3**	+	126.03	126.9	109.0	25	8	2.19	1.80–2.80
**4**	−	374.12	373.0	122.9	50	15	2.48	2.00–3.00
**5**	−	496.16	495.0	136.9	70	36	3.53	3.10–4.10
**6**	+	390.15	391.1	228.9	12	8	4.17	3.70–4.70
**7**	−	388.14	446.9	224.9	26	12	4.20	3.80–4.80
**8**	−	480.16	478.9	448.9	60	5	4.76	4.30–5.30
**9**	+	480.31	481.2	445.1	24	12	5.25	4.80–5.80
**10**	+	624.21	625.1	163.0	16	28	5.32	4.90–5.90
**11**	+	542.16	543.1	211.0	16	14	5.92	5.50–6.50
**12**	−	584.19	583.0	553.0	70	5	7.99	7.58–8.58
**13**	+	166.06	166.9	42.9	16	16	8.98	8.60–8.60
**14**	+	514.37	515.3	97.0	16	20	15.53	15.10–16.10

**Table 2:** Linear range, regression equation, *r*^2^, LOD, and LOQ of each marker analyte for UPLC–MS/MS MRM analysis (*n* = 3).

Analyte	Linear Range (µg/L)	Regression Equation ^a^ *y* = a*x* + b	*r* ^2^	LOD (µg/L)	LOQ (µg/L)
**1**	35.00–1400.00	*y* = 197.40*x* + 715.28	0.9977	0.03	0.10
**2**	900.00–36,000.00	*y* = 16.10*x* − 5046.77	0.9968	3.07	9.22
**3**	29.50–1180.00	*y* = 688.83*x* + 12805.60	0.9925	5.13	15.40
**4**	30.00–1200.00	*y* = 32.67*x* − 209.54	0.9997	0.01	0.03
**5**	35.00–1400.00	*y* = 47.69*x* − 716.09	0.9979	0.65	1.95
**6**	80.00–3200.00	*y* = 15.21*x* − 472.66	0.9962	2.63	7.88
**7**	3.00–120.00	*y* = 117.72*x* − 220.62	0.9988	0.27	0.81
**8**	140.00–5600.00	*y* = 17.49*x* − 260.54	0.9976	1.06	3.18
**9**	22.50–900.00	*y* = 207.44*x* + 2194.41	0.9954	0.10	0.31
**10**	11.00–440.00	*y* = 55.33*x* − 247.39	0.9992	0.26	0.79
**11**	15.00–600.00	*y* = 97.19*x* − 310.13	0.9993	0.18	0.53
**12**	30.00–1200.00	*y* = 19.28*x* − 61.53	0.9996	0.29	0.88
**13**	200.00–8000.00	*y* = 777.86*x* − 987.75	0.9983	0.01	0.02
**14**	0.60–24.00	*y* = 249.82*x* + 15.06	0.9905	0.18	0.54

^a^*y* and *x* represent the respective peak areas at different concentrations (µg/L) of each reference standard compound. Allantoin (**1**), gallic acid (**2**), 5-(hydroxymethyl)furfural (**3**), geniposidic acid (**4**), oxypaeoniflorin (**5**), loganin (**6**), geniposide (**7**), paeoniflorin (**8**), ecdysterone (**9**), verbascoside (**10**), cornuside (**11**), benzoylpaeoniflorin (**12**), paeonol (**13**), and alisol B acetate (**14**).

**Table 3:** Recovery (%) test of 14 markers using the UPLC–MS/MS MRM method.

Analyte	Spiked Amount (µg/L)	Found Amount (µg/L)	Recovery (%) ^a^	SD	CV (%)
**1**	733.35	674.53	91.98	5.11	0.76
	883.35	808.81	91.56	17.10	2.11
	1033.35	941.72	91.13	27.21	2.89
**2**	11,206.90	13,407.94	119.64	196.05	1.46
	21,206.90	23,164.32	109.23	74.13	0.32
	31,206.90	32,056.30	102.72	80.22	0.25
**3**	504.65	451.45	89.46	24.17	5.35
	799.65	932.21	116.58	45.37	4.87
	1094.65	1279.31	116.87	41.54	3.25
**4**	471.05	514.32	109.19	8.33	1.62
	771.05	815.69	105.79	34.82	4.27
	1071.05	1140.28	106.46	12.50	1.10
**5**	703.75	760.04	108.00	9.89	1.30
	1053.75	1171.82	111.21	17.59	1.50
	1403.75	1622.10	115.56	28.16	1.74
**6**	1634.27	1712.97	104.82	22.09	1.29
	2434.27	2517.14	103.40	102.05	4.05
	3234.27	3402.03	105.19	130.06	3.82
**7**	79.57	79.23	99.58	0.68	0.86
	119.57	119.56	99.99	4.52	3.78
	159.57	164.90	103.34	0.48	0.29
**8**	2813.95	3274.32	116.36	45.32	1.38
	4213.95	4667.37	110.76	110.34	2.36
	5613.95	5962.55	106.21	171.33	2.87
**9**	263.27	282.25	107.21	8.34	2.95
	488.27	521.97	106.90	23.10	4.43
	713.27	699.62	98.09	63.32	9.05
**10**	237.67	233.55	98.27	3.09	1.33
	347.67	316.93	91.16	12.72	4.01
	457.67	378.21	82.64	4.97	1.31
**11**	278.00	252.58	90.86	5.41	2.14
	428.00	354.77	82.89	13.05	3.68
	578.00	443.05	76.65	7.83	1.77
**12**	588.50	681.32	115.77	11.10	1.63
	888.50	1037.52	116.77	32.56	3.14
	1188.50	1420.09	119.49	19.36	1.35
**13**	3944.38	4257.84	107.95	31.19	0.73
	5944.38	6278.03	105.61	130.85	2.08
	7944.38	8211.59	103.36	25.12	0.31
**14**	12.48	13.01	104.22	0.32	2.42
	24.24	24.40	100.64	0.84	3.45
	39.12	35.72	91.32	1.02	2.86

^a^ Recovery (%) = (found amount/spiked amount) × 100. Allantoin (**1**), gallic acid (**2**), 5-(hydroxymethyl)furfural (**3**), geniposidic acid (**4**), oxypaeoniflorin (**5**), loganin (**6**), geniposide (**7**), paeoniflorin (**8**), ecdysterone (**9**), verbascoside (**10**), cornuside (**11**), benzoylpaeoniflorin (**12**), paeonol (**13**), and alisol B acetate (**14**).

**Table 4:** Precision results of the developed UPLC–MS/MS MRM method using 14 markers (*n* = 3).

Analyte	Conc. (µg/L)	Intra-Day			Inter-Day		
		Observed Conc. (µg/L)	Precision (CV, %)	Accuracy (%)	Observed Conc. (µg/L)	Precision (CV, %)	Accuracy (%)
**1**	175.00	160.66	1.51	91.80	177.43	9.91	101.39
	350.00	373.88	1.82	106.82	374.08	1.90	106.88
	700.00	725.69	1.87	103.67	710.82	5.77	101.55
**2**	9000.00	9593.69	1.72	106.60	8130.41	2.22	90.34
	18,000.00	16,350.19	2.65	90.83	17,764.99	2.39	98.69
	27,000.00	26,606.21	0.72	98.54	27,000.40	4.17	100.00
**3**	1475.00	1506.92	2.24	102.16	1524.46	7.64	103.35
	2950.00	2997.51	8.55	101.61	3165.14	8.74	107.29
	5900.00	5759.22	3.03	97.61	6098.16	7.40	103.36
**4**	150.00	158.36	3.99	105.57	153.14	5.12	102.10
	300.00	315.64	2.48	105.21	310.71	2.91	103.57
	600.00	609.36	2.25	101.56	604.61	2.51	100.77
**5**	175.00	166.42	4.69	95.10	167.33	1.72	95.62
	350.00	341.86	7.72	97.68	341.00	2.68	97.43
	700.00	677.57	1.61	96.80	672.49	1.21	96.07
**6**	400.00	405.00	3.49	101.25	399.32	6.04	99.83
	800.00	803.91	1.60	100.49	818.92	6.90	102.36
	1600.00	1598.20	1.30	99.89	1545.80	4.49	96.61
**7**	15.00	15.34	6.91	102.29	14.89	5.57	99.24
	30.00	31.21	2.17	104.02	30.45	6.04	101.50
	60.00	60.71	2.61	101.18	59.19	1.53	98.66
**8**	700.00	655.24	1.03	93.61	711.55	7.71	101.65
	1400.00	1493.31	0.72	106.67	1502.72	0.72	107.34
	2800.00	2874.30	0.84	102.65	2795.28	5.05	99.83
**9**	112.50	106.09	2.50	94.31	120.33	3.36	106.96
	225.00	237.78	2.02	105.68	245.79	1.85	109.24
	450.00	456.85	3.31	101.52	449.38	7.08	99.86
**10**	75.00	77.81	2.13	103.75	73.52	3.86	98.02
	150.00	143.57	2.50	95.71	153.20	8.10	102.13
	300.00	300.86	0.17	100.29	298.44	1.18	99.48
**11**	75.00	68.19	0.73	90.92	77.19	5.71	102.92
	150.00	143.60	1.24	95.73	155.04	2.65	103.36
	300.00	290.52	2.16	96.84	300.37	2.35	100.12
**12**	150.00	157.77	1.47	105.18	156.51	0.19	104.34
	300.00	316.03	2.60	105.34	306.18	0.74	102.06
	600.00	612.99	0.74	102.17	595.89	1.12	99.32
**13**	1000.00	957.04	1.14	95.70	1019.41	7.79	101.94
	2000.00	2070.86	1.21	103.54	2127.56	1.21	106.38
	4000.00	4097.28	0.98	102.43	4010.78	3.80	100.27
**14**	6.00	5.65	5.98	94.22	5.79	5.87	96.56
	12.00	12.01	5.23	100.06	11.33	6.49	94.39
	18.00	17.94	0.46	99.67	18.18	2.55	101.02

Allantoin (**1**), gallic acid (**2**), 5-(hydroxymethyl)furfural (**3**), geniposidic acid (**4**), oxypaeoniflorin (**5**), loganin (**6**), geniposide (**7**), paeoniflorin (**8**), ecdysterone (**9**), verbascoside (**10**), cornuside (**11**), benzoylpaeoniflorin (**12**), paeonol (**13**), and alisol B acetate (**14**).

**Table 5:** Contents of compounds **1**–**14** in GSH samples (*n* = 3).

Analyte	GSH–1 ^a^			GSH–2			Source ^b^
	Mean (mg/kg)	SD	CV (%)	Mean (mg/kg)	SD	CV (%)	
**1**	1162.07	24.00	2.07	1183.52	9.42	0.80	DJ
**2**	2404.19	62.35	2.59	1152.41	13.78	1.20	CO
**3**	3489.64	214.43	6.14	4126.28	393.28	9.53	RG
**4**	340.71	2.53	0.74	573.31	0.94	0.16	PA
**5**	704.68	9.47	1.34	464.09	1.38	0.30	PS
**6**	1661.91	82.90	4.99	1521.40	86.36	5.68	CO
**7**	78.81	3.04	3.86	80.72	2.06	2.56	PA
**8**	2816.66	78.32	2.78	2108.67	80.24	3.81	PS
**9**	76.23	1.07	1.41	399.93	6.53	1.63	AB
**10**	254.32	6.24	2.45	202.42	4.76	2.35	PA
**11**	254.97	4.38	1.72	193.73	2.88	1.49	CO
**12**	574.69	17.90	3.12	348.49	2.87	0.82	PS
**13**	3873.26	48.08	1.24	1087.89	5.88	0.54	PS
**14**	6.94	0.48	6.97	15.97	0.38	2.40	AO

^a^ GSH–1 and GSH–2 samples were prepared by the Korea Institute of Oriental Medicine and Wonkwang University, respectively. ^b^ DJ: *D. japonica*; CO: *C. officinalis*, RG: *R. glutinosa*, PA: *P. asiatica*, PS: *P. suffruticosa*, AB: *A. bidentate*, and AO: *A. orientale*. Allantoin (**1**), gallic acid (**2**), 5-(hydroxymethyl)furfural (**3**), geniposidic acid (**4**), oxypaeoniflorin (**5**), loganin (**6**), geniposide (**7**), paeoniflorin (**8**), ecdysterone (**9**), verbascoside (**10**), cornuside (**11**), benzoylpaeoniflorin (**12**), paeonol (**13**), and alisol B acetate (**14**).

## Data Availability

All data are available in this article.

## Supplementary Materials

The following supporting information can be downloaded at: https://www.mdpi.com/article/10.3390/molecules27123890/s1, Figure S1: Total ion chromatograms of the 21 investigated standard component mixtures (A) and GSH sample (B) obtained using the UPLC–MS/MS MRM method in the positive and negative ion modes. Allantoin (**a**), gallic acid (**b**), 5-(hydroxymethyl)furfural (**c**), geniposidic acid (**d**), morroniside (**e**), oxypaeoniflorin (**f**), loganin (**g**), geniposide (**h**), paeoniflorin (**i**), ecdysterone (**j**), verbascoside (**k**), cornuside (**l**), benzoic acid (**m**), benzoylpaeoniflorin (**n**), paeonol (**o**), dioscin (**p**), polyporenic acid C (**q**), sweroside (**r**), alisol B (**s**), alisol B acetate (**t**), and pachymic acid (**u**). Figure S2: Extracted ion chromatograms for blank of each marker compound. Figure S3: Q1 (A) and Q3 (B) mass spectra of 14 marker components. Allantoin (**1**), gallic acid (**2**), 5-(hydroxymethyl)furfural (**3**), geniposidic acid (**4**), oxypaeoniflorin (**5**), loganin (**6**), geniposide (**7**), paeoniflorin (**8**), ecdysterone (**9**), verbascoside (**10**), cornuside (**11**), benzoylpaeoniflorin (**12**), paeonol (**13**), and alisol B acetate (**14**). Figure S4: Calibration curves and residuals of 14 marker components. A; allantoin, B; gallic acid, C; 5-(hydroxymethyl)furfural, D; geniposidic acid, E; oxypaeoniflorin, F; loganin, G; geniposide, H; paeoniflorin, I; ecdysterone, J; verbascoside, K; cornuside, L; benzoylpaeoniflorin, M; paeonol, and N; alisol B acetate. Figure S5: Total ion chromatogram of LOD using the UPLC–MS/MS MRM method in the positive and negative ion modes. Figure S6: Extracted ion chromatograms of each marker compound (A) and GSH sample (B) using the UPLC–MS/MS MRM method in the positive and negative ion modes. Figure S7: Chemical structures of the 14 marker components in GSH. Table S1: UPLC–MS/MS conditions for simultaneous analysis of the 14 marker components in GSH samples. Table S2: Repeatability of retention time of the 14 marker analytes in the developed UPLC–MS/MS MRM assay (n = 6). Table S3: Composition of GSH.

## Sample Availability

Samples of the compounds are available from the authors.
