# Supplementary material for: Simultaneous Determination of Fourteen Marker Compounds in the Traditional Herbal Prescription, Geumgwesingihwan, Using Ultra-Performance Liquid Chromatography–Tandem Mass Spectrometry
Source: Molecules. 2022 Jun 17;27(12):3890. doi: 10.3390/molecules27123890 (PMC9227478; doi:10.3390/molecules27123890)
Supplement: Supplementary file 1 [file molecules-27-03890-s001.zip › molecules-1778679-supplementary.pdf]

Table S1. UPLC–MS/MS conditions for simultaneous analysis of the 14 marker components in GSH samples.

**UPLC conditions**				**MS/MS conditions**	
LC system	Acquity UPLC H-Class			MS system	Xevo TQ-S micro
Column	Acquity UPLC BEH C_{18} column (2.1 mm × 100 mm, 1.7 μm)			MS software	MassLynx (version 4.2)
Column temperature	40 °C			Ionization source	ESI
Sample temperature	5 °C			Ionization mode	Positive/Negative
Injection volume	2.0 μL			Acquisition mode	MRM
Flow rate	0.3 mL/min			Capillary voltage	3.3 kV
Mobile phase A	0.1% (v/v) Acetic acid in distilled water			Cone gas flow	80 L/h
Mobile phase B	0.1% (v/v) Acetic acid in acetonitrile			Desolvation gas flow	600 L/h
Gradient elution	Time (min)	A (%)	B (%)	Desolvation temperature	300 °C
	0.00	95	5	Source temperature	150 °C
	11.43	40	60		
	14.29	5	95		
	15.71	5	95		
	17.14	95	5		
	20.00	95	5		

Table S2. Repeatability of retention time of the 14 marker analytes in the developed UPLC–MS/MS MRM assay (n = 6).

Analyte	Retention time						Mean	SD	CV (%)
	1	2	3	4	5	6			
**1**	0.79	0.79	0.80	0.79	0.79	0.80	0.79	0.01	0.65
**2**	1.43	1.46	1.48	1.48	1.49	1.49	1.47	0.02	1.57
**3**	2.15	2.18	2.19	2.21	2.21	2.21	2.19	0.02	1.10
**4**	2.43	2.47	2.47	2.50	2.50	2.50	2.48	0.03	1.12
**5**	3.48	3.52	3.53	3.55	3.55	3.54	3.53	0.03	0.75
**6**	4.14	4.16	4.17	4.18	4.18	4.17	4.17	0.02	0.36
**7**	4.17	4.19	4.20	4.21	4.21	4.21	4.20	0.02	0.38
**8**	4.72	4.75	4.76	4.77	4.77	4.76	4.76	0.02	0.39
**9**	5.25	5.25	5.25	5.25	5.26	5.25	5.25	0.00	0.08
**10**	5.29	5.32	5.33	5.33	5.33	5.33	5.32	0.02	0.30
**11**	5.89	5.91	5.92	5.93	5.92	5.93	5.92	0.02	0.25
**12**	7.98	7.99	7.99	7.99	7.99	7.99	7.99	0.00	0.05
**13**	8.92	8.98	9.00	9.00	9.00	9.00	8.98	0.03	0.36
**14**	15.52	15.53	15.53	15.53	15.53	15.53	15.53	0.00	0.03

Allantoin (**1**), gallic acid (**2**), 5-(hydroxymethyl)furfural (**3**), geniposidic acid (**4**), oxypaeoniflorin (**5**), loganin (**6**), geniposide (**7**), paeoniflorin (**8**), ecdysterone (**9**), verbascoside (**10**), cornuside (**11**), benzoylpaeoniflorin (**12**), paeonol (**13**), and alisol B acetate (**14**).

Table S3. Composition of GSH.

Botanical name	Scientific name	Family	Used part	Origin	Amount (g)	Ratio (%)
Rehmanniae Radix Preparata	*Rehmannia glutinosa* (Gaertn.) DC.	Plantaginaceae	Root	Jeongeup, Korea	1481.4	29.7
Dioscoreae Rhizoma	*Dioscorea japonica* Thunb.	Dioscoreaceae	Rhizome	Korea	740.7	14.8
Corni Fructus	*Cornus officinalis* Siebold & Zucc.	Cornaceae	Fruit	Gurye, Korea	740.7	14.8
Poria Sclerotium	*Poria cocos* Wolf	Polyporaceae	Sclerotium	Binghwa, Korea	555.6	11.1
Moutan Radicis Cortex	*Paeonia suffruticosa* Andrews	Paeoniaceae	Root bark	Imsil, Korea	555.6	11.1
Alisamatis Rhizoma	*Alisma plantago-aquatica* subsp. *orientale* (Sam.) Sam.	Alismataceae	Rhizome	Imsil, Korea	555.6	11.1
Achyranthis Radix	*Achyranthes bidentate* Blume	Amaranthaceae	Root	Hwasun, Korea	185.2	3.7
Plantaginis Semen	*Plantago asiatica* L.	Plantaginaceae	Seed	China	185.2	3.7
				Total	5000.0	100.0

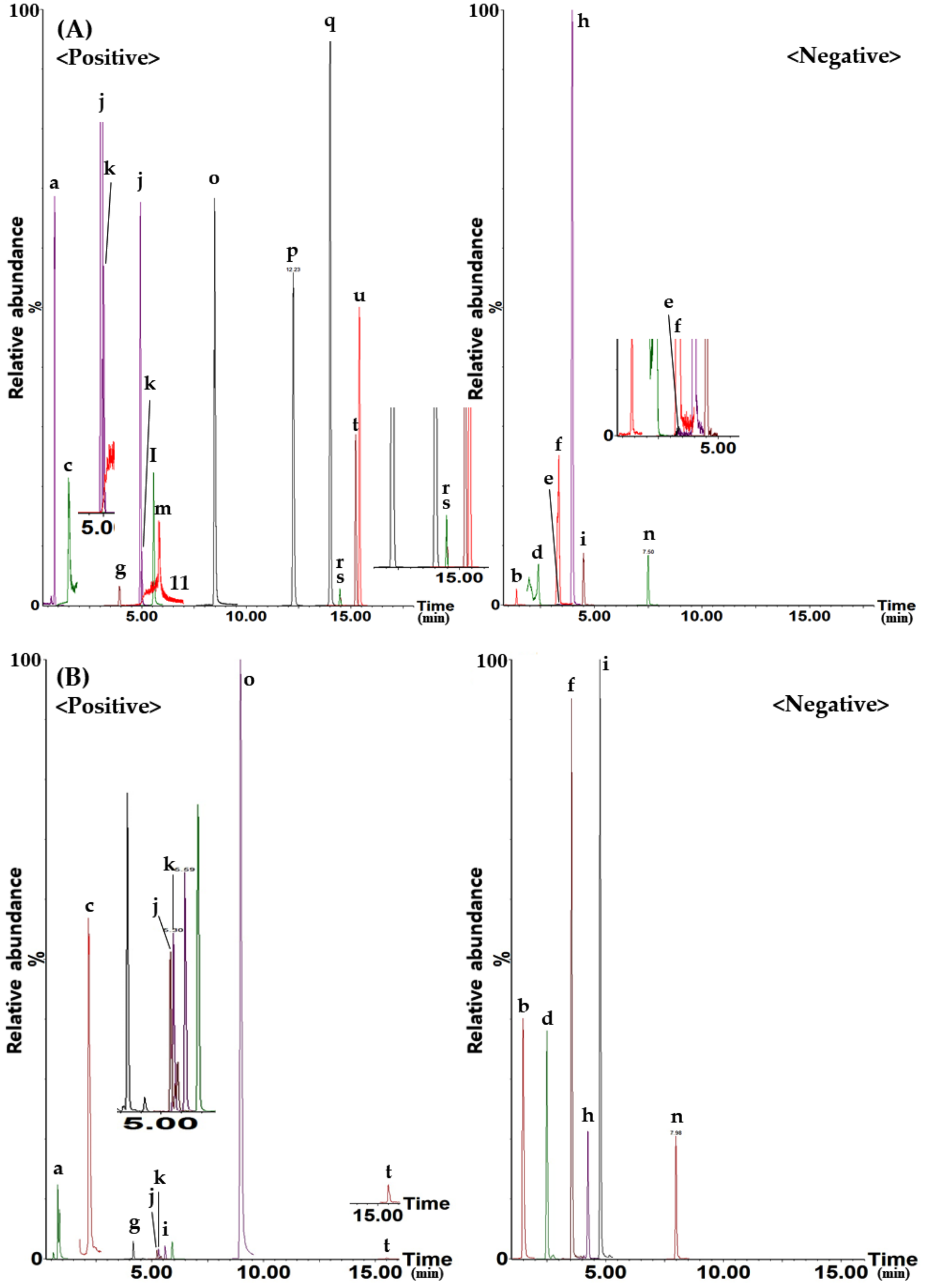
(A)
<Positive>
<Negative>
(B)
<Positive>
<Negative>
Relative abundance
%
100
0
5.00
10.00
15.00
Time
(min)
a
b
c
d
e
f
g
h
i
j
k
l
m
n
o
p
q
r
s
t
u
11

Figure S1. Total ion chromatograms of the 21 investigated standard component mixtures (A) and GSH sample (B) obtained using the UPLC–MS/MS MRM method in the positive and negative ion modes. Allantoin (**a**), gallic acid (**b**), 5-(hydroxymethyl)furfural (**c**), geniposidic acid (**d**), morroniside (**e**), oxypaeoniflorin (**f**), loganin (**g**), geniposide (**h**), paeoniflorin (**i**), ecdysterone (**j**), verbascoside (**k**), cornuside (**l**), benzoic acid (**m**), benzoylpaeoniflorin (**n**), paeonol (**o**), dioscin (**p**), polyporenic acid C (**q**), sweroside (**r**), alisol B (**s**), alisol B acetate (**t**), and pachymic acid (**u**).

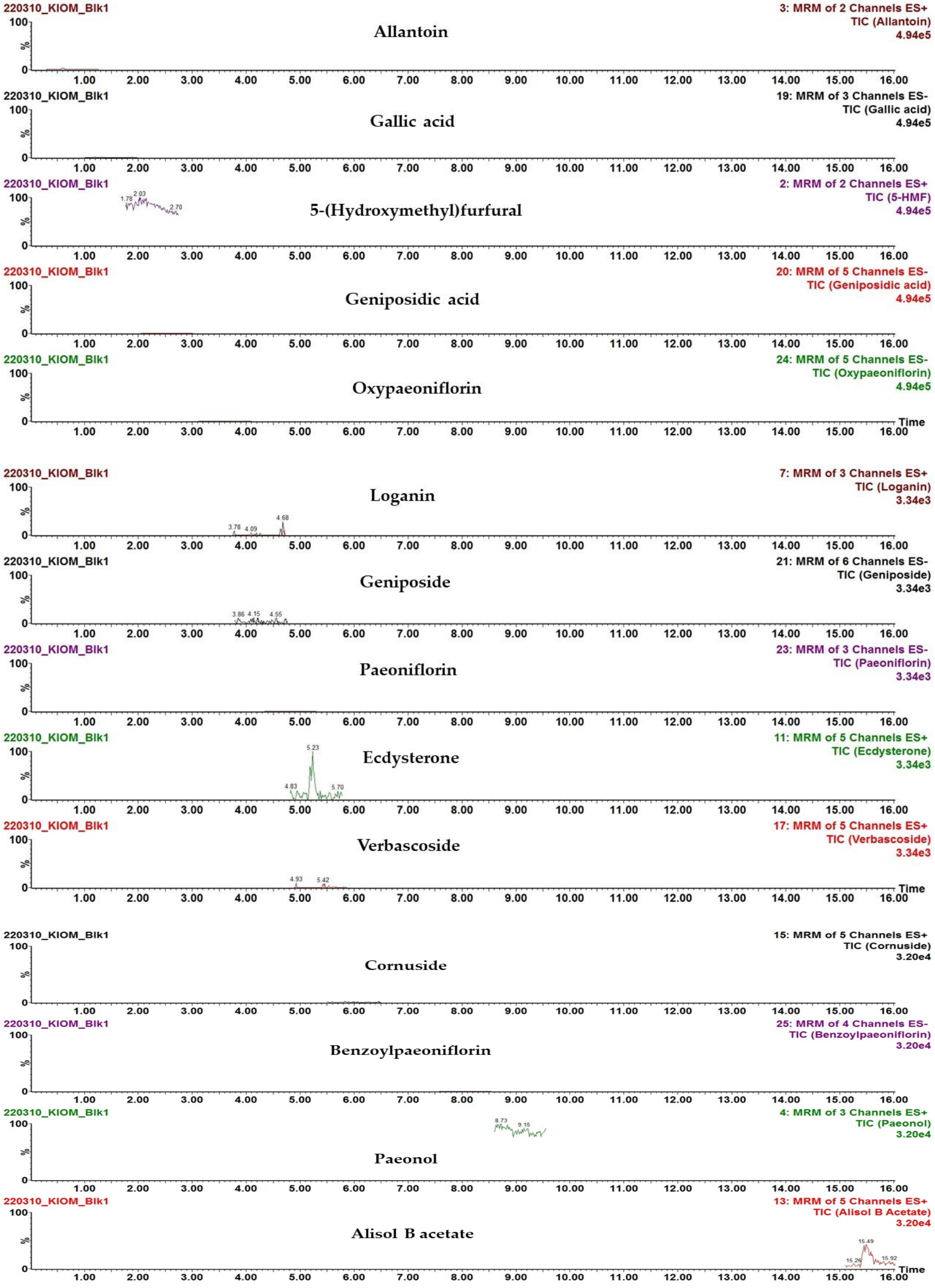

Figure S2. Extracted ion chromatograms for blank of each marker compound.

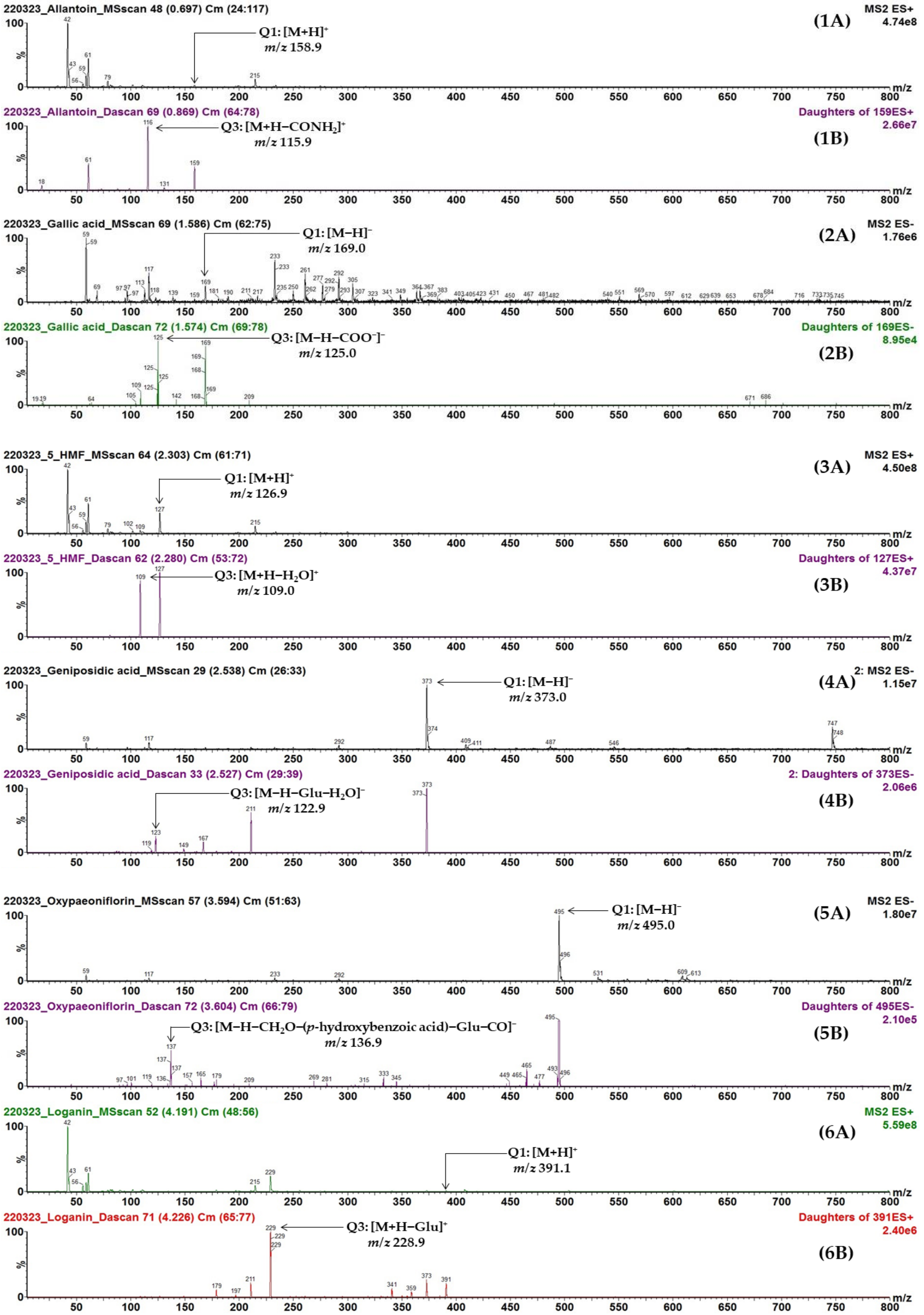
220323_Allantoin_MSscan 48 (0.697) Cm (24:117)
MS2 ES+
4.74e8
(1A)
Q1: [M+H]+
m/z 158.9
220323_Allantoin_Dascan 69 (0.869) Cm (64:78)
Daughters of 159ES+
2.66e7
(1B)
Q3: [M+H−CONH2]+
m/z 115.9
220323_Gallic acid_MSscan 69 (1.586) Cm (62:75)
MS2 ES-
1.76e6
(2A)
Q1: [M−H]−
m/z 169.0
220323_Gallic acid_Dascan 72 (1.574) Cm (69:78)
Daughters of 169ES-
8.95e4
(2B)
Q3: [M−H−COO−]−
m/z 125.0
220323_5_HMF_MSscan 64 (2.303) Cm (61:71)
MS2 ES+
4.50e8
(3A)
Q1: [M+H]+
m/z 126.9
220323_5_HMF_Dascan 62 (2.280) Cm (53:72)
Daughters of 127ES+
4.37e7
(3B)
Q3: [M+H−H2O]+
m/z 109.0
220323_Geniposidic acid_MSscan 29 (2.538) Cm (26:33)
2: MS2 ES-
1.15e7
(4A)
Q1: [M−H]−
m/z 373.0
220323_Geniposidic acid_Dascan 33 (2.527) Cm (29:39)
2: Daughters of 373ES-
2.06e6
(4B)
Q3: [M−H−Glu−H2O]−
m/z 122.9
220323_Oxypaeoniflorin_MSscan 57 (3.594) Cm (51:63)
MS2 ES-
1.80e7
(5A)
Q1: [M−H]−
m/z 495.0
220323_Oxypaeoniflorin_Dascan 72 (3.604) Cm (66:79)
Daughters of 495ES-
2.10e5
(5B)
Q3: [M−H−CH2O−(p-hydroxybenzoic acid)−Glu−CO]−
m/z 136.9
220323_Loganin_MSscan 52 (4.191) Cm (48:56)
MS2 ES+
5.59e8
(6A)
Q1: [M+H]+
m/z 391.1
220323_Loganin_Dascan 71 (4.226) Cm (65:77)
Daughters of 391ES+
2.40e6
(6B)
Q3: [M+H−Glu]+
m/z 228.9
m/z
%

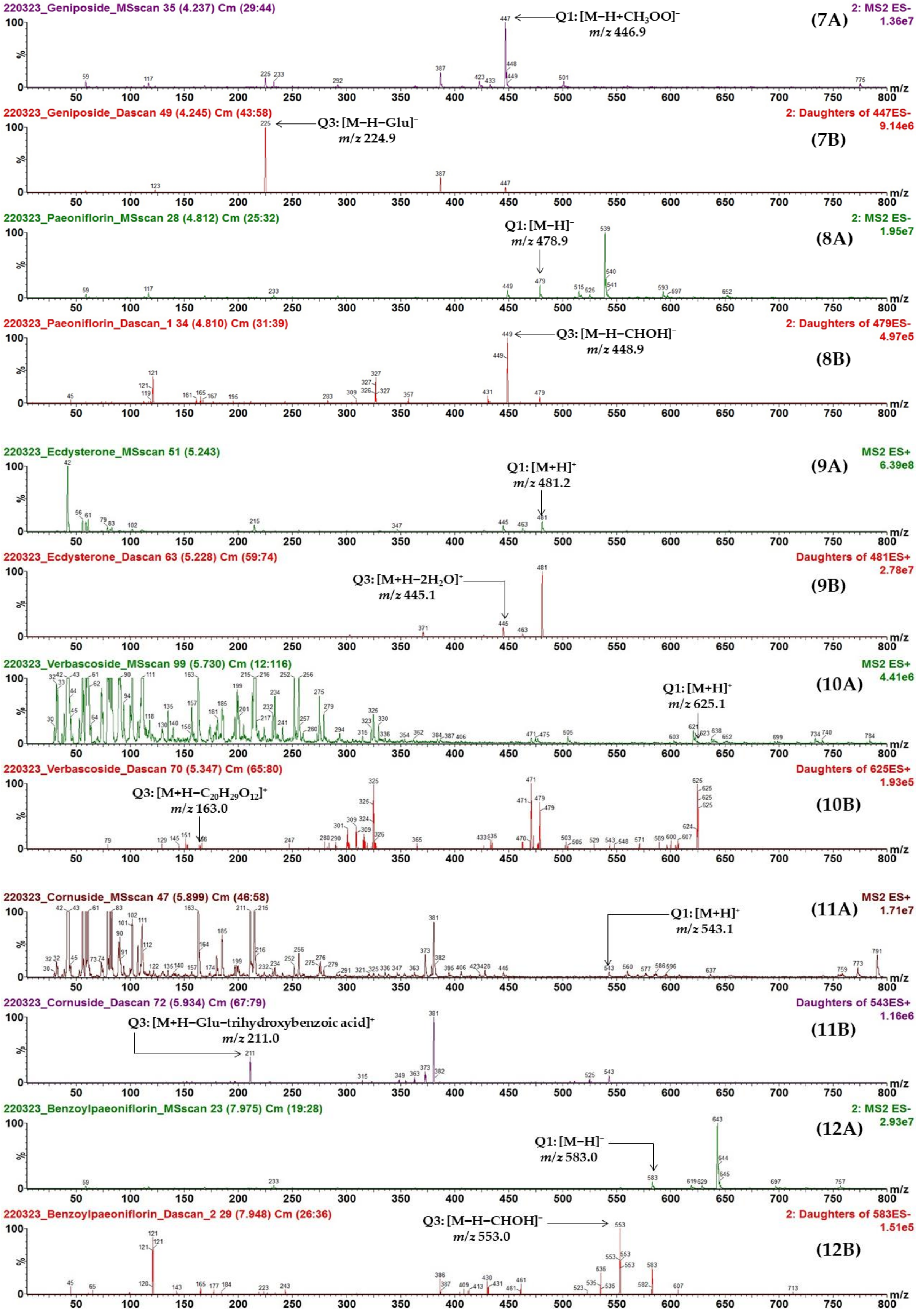
220323_Geniposide_MSscan 35 (4.237) Cm (29:44)
2: MS2 ES-
1.36e7
(7A)
Q1: [M−H+CH3OO]−
m/z 446.9
220323_Geniposide_Dascan 49 (4.245) Cm (43:58)
2: Daughters of 447ES-
9.14e6
(7B)
Q3: [M−H−Glu]−
m/z 224.9
220323_Paeoniflorin_MSscan 28 (4.812) Cm (25:32)
2: MS2 ES-
1.95e7
(8A)
Q1: [M−H]−
m/z 478.9
220323_Paeoniflorin_Dascan_1 34 (4.810) Cm (31:39)
2: Daughters of 479ES-
4.97e5
(8B)
Q3: [M−H−CHOH]−
m/z 448.9
220323_Ecdysterone_MSscan 51 (5.243)
MS2 ES+
6.39e8
(9A)
Q1: [M+H]+
m/z 481.2
220323_Ecdysterone_Dascan 63 (5.228) Cm (59:74)
Daughters of 481ES+
2.78e7
(9B)
Q3: [M+H−2H2O]+
m/z 445.1
220323_Verbascoside_MSscan 99 (5.730) Cm (12:116)
MS2 ES+
4.41e6
(10A)
Q1: [M+H]+
m/z 625.1
220323_Verbascoside_Dascan 70 (5.347) Cm (65:80)
Daughters of 625ES+
1.93e5
(10B)
Q3: [M+H−C20H29O12]+
m/z 163.0
220323_Cornuside_MSscan 47 (5.899) Cm (46:58)
MS2 ES+
1.71e7
(11A)
Q1: [M+H]+
m/z 543.1
220323_Cornuside_Dascan 72 (5.934) Cm (67:79)
Daughters of 543ES+
1.16e6
(11B)
Q3: [M+H−Glu−trihydroxybenzoic acid]+
m/z 211.0
220323_Benzoylpaeoniflorin_MSscan 23 (7.975) Cm (19:28)
2: MS2 ES-
2.93e7
(12A)
Q1: [M−H]−
m/z 583.0
220323_Benzoylpaeoniflorin_Dascan_2 29 (7.948) Cm (26:36)
2: Daughters of 583ES-
1.51e5
(12B)
Q3: [M−H−CHOH]−
m/z 553.0
m/z
%

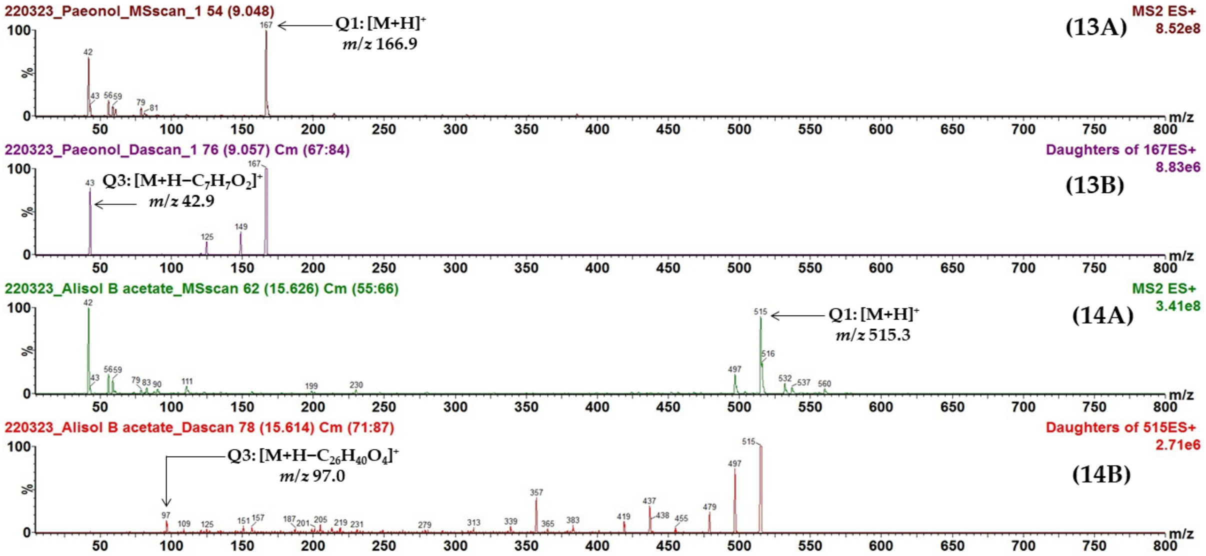

Figure S3. Q1 (A) and Q3 (B) mass spectra of 14 marker components. Allantoin (**1**), gallic acid (**2**), 5-(hydroxymethyl)furfural (**3**), geniposidic acid (**4**), oxypaeoniflorin (**5**), loganin (**6**), geniposide (**7**), paeoniflorin (**8**), ecdysterone (**9**), verbascoside (**10**), cornuside (**11**), benzoylpaeoniflorin (**12**), paeonol (**13**), and alisol B acetate (**14**).

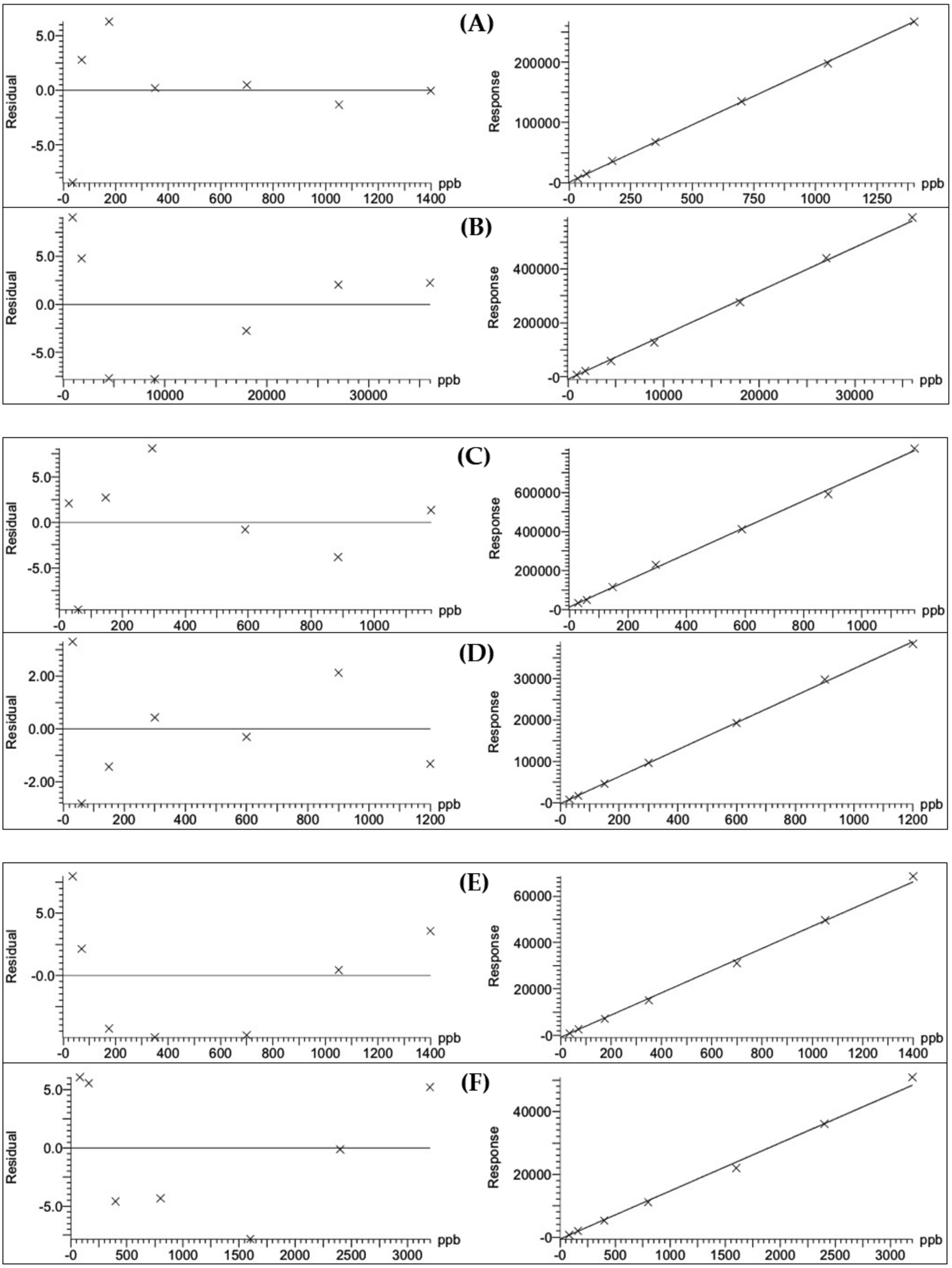
(A)
Residual
Response
ppb
(B)
Residual
Response
ppb
(C)
Residual
Response
ppb
(D)
Residual
Response
ppb
(E)
Residual
Response
ppb
(F)
Residual
Response
ppb

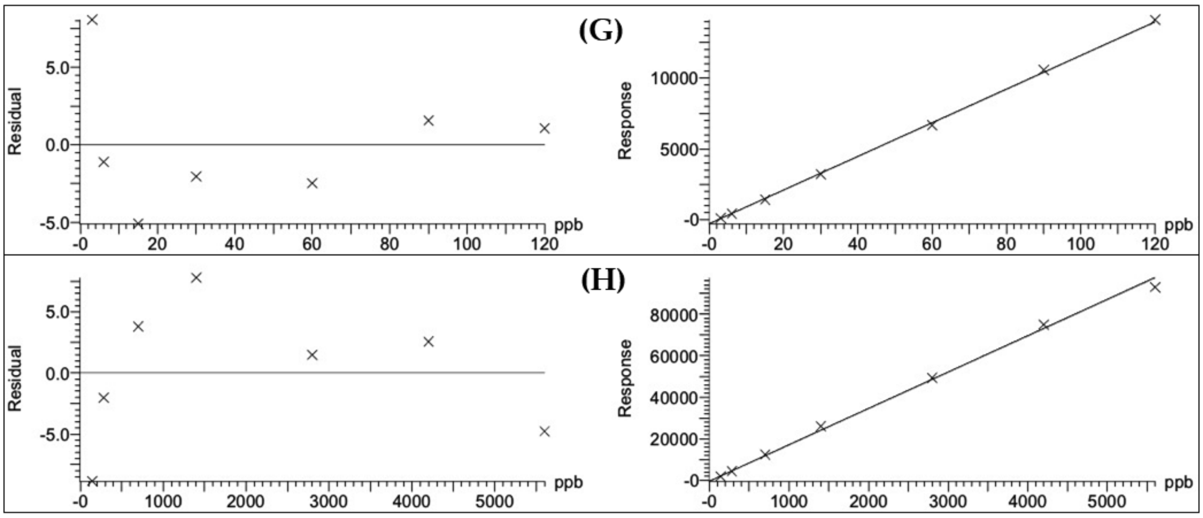
(G)
(H)
Residual
Response
ppb

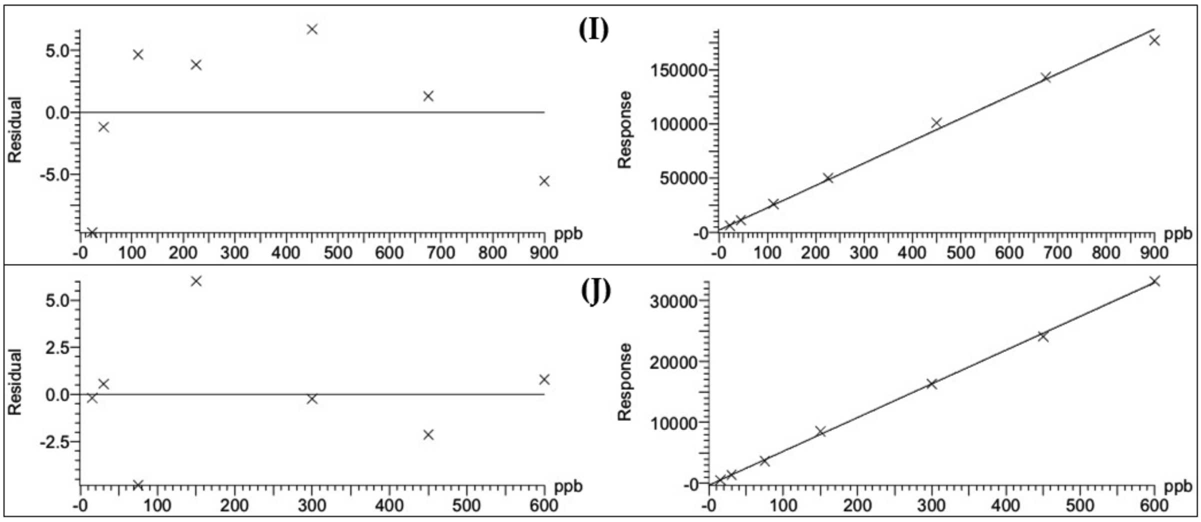
(I)
(J)
Residual
Response
ppb

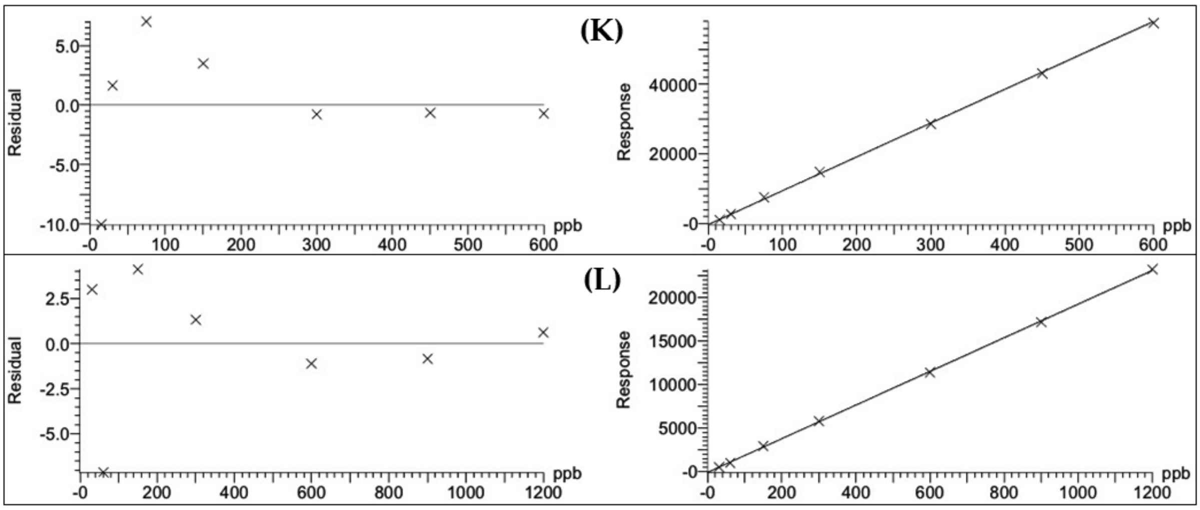
(K)
(L)
Residual
Response
ppb

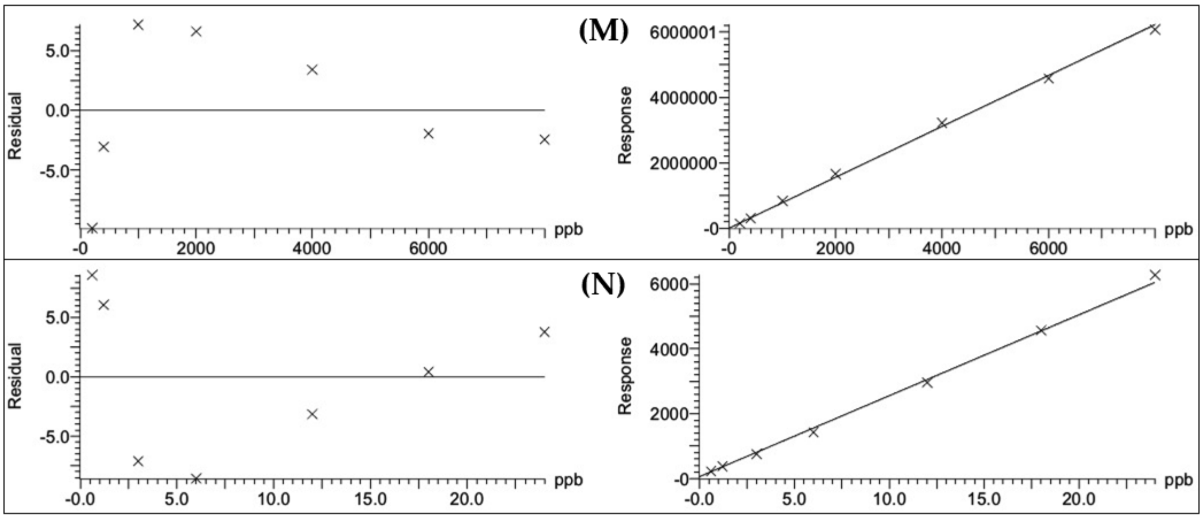

Figure S4. Calibration curves and residuals of 14 marker components. A; allantoin, B; gallic acid, C; 5-(hydroxymethyl)furfural, D; geniposidic acid, E; oxypaeoniflorin, F; loganin, G; geniposide, H; paeoniflorin, I; ecdysterone, J; verbascoside, K; cornuside, L; benzoylpaeoniflorin, M; paeonol, and N; alisol B acetate.

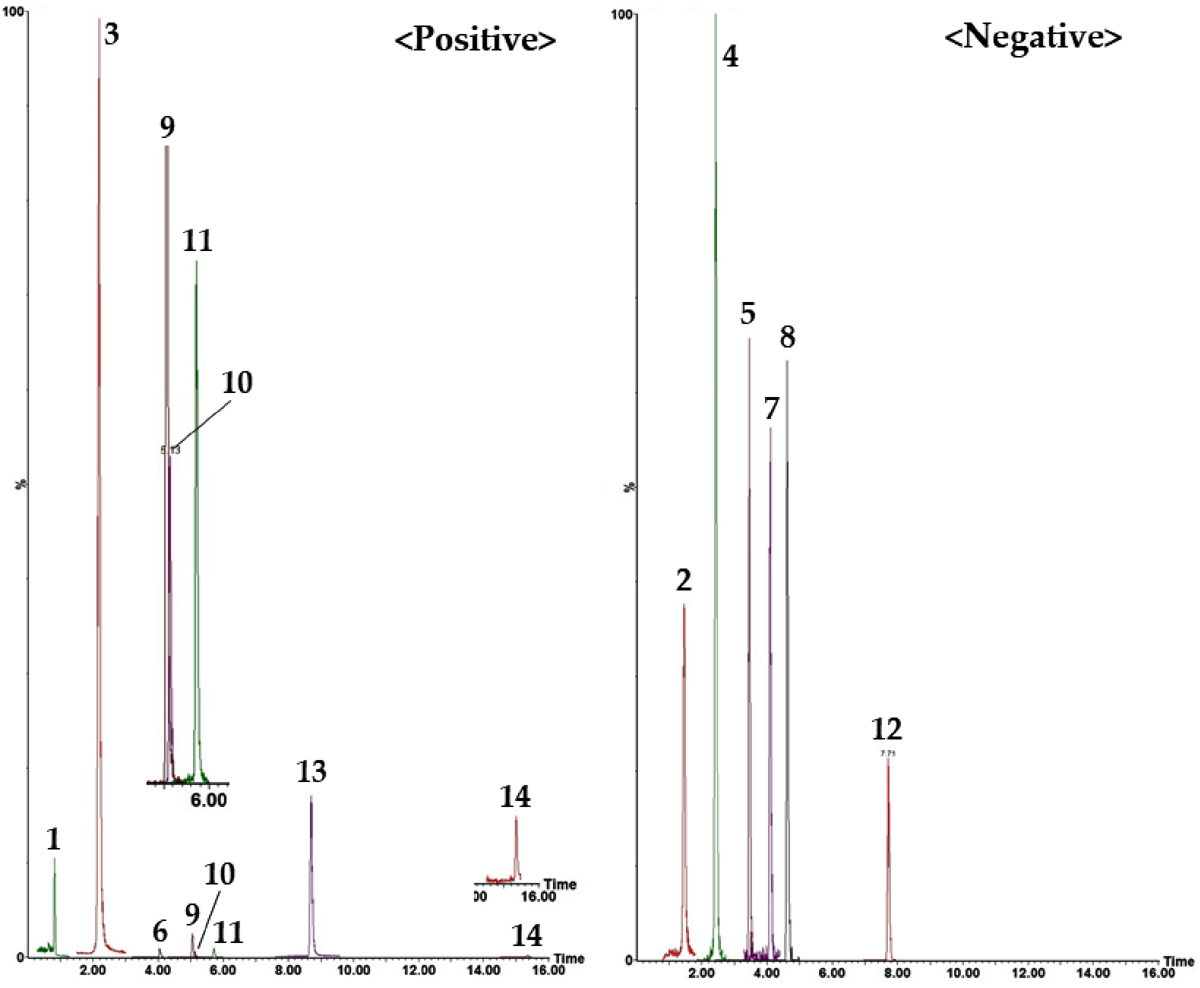

Figure S5. Total ion chromatogram of LOD using the UPLC–MS/MS MRM method in the positive and negative ion modes.

(A)

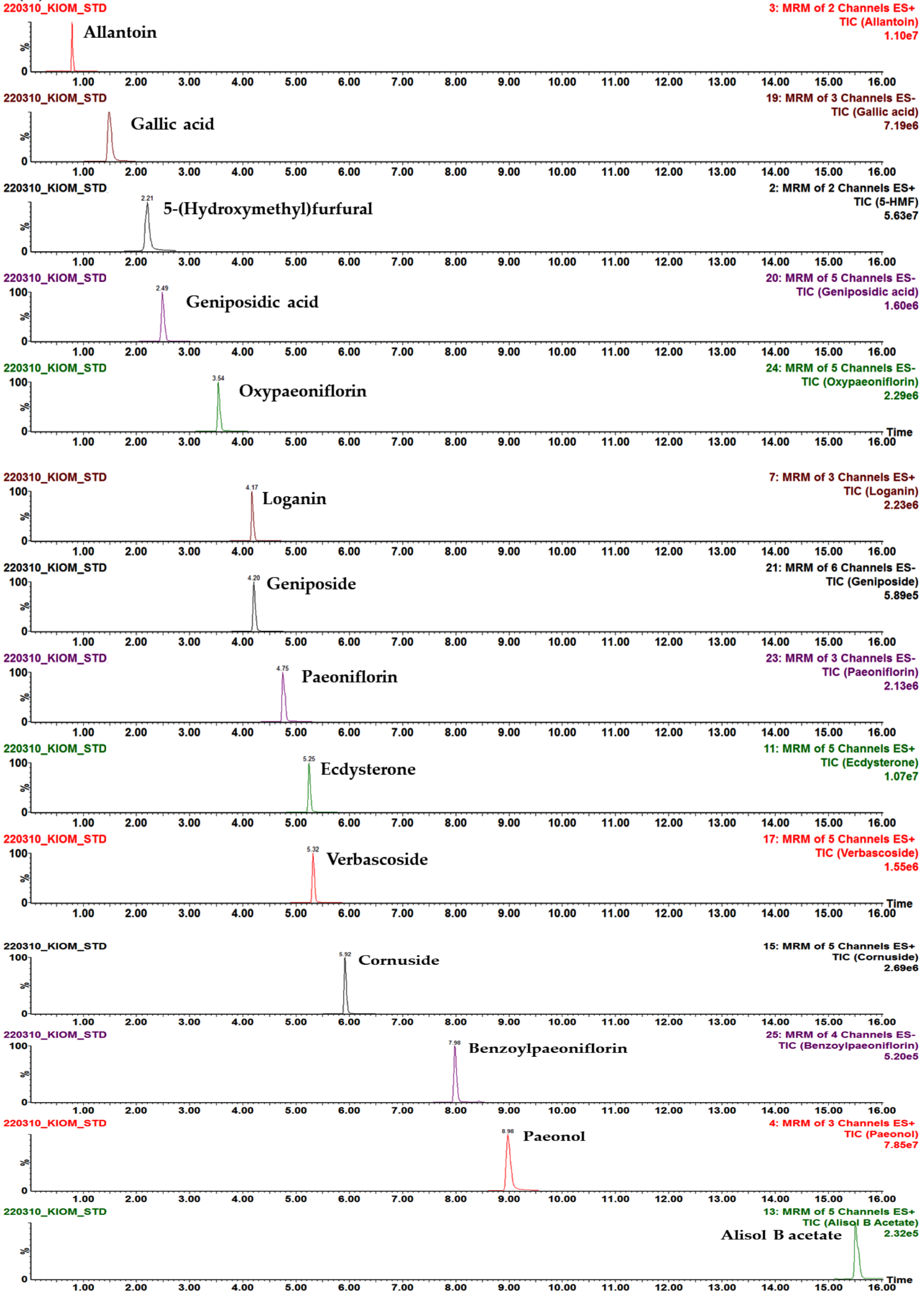
220310_KIOM_STD
3: MRM of 2 Channels ES+
TIC (Allantoin)
1.10e7
Allantoin
19: MRM of 3 Channels ES-
TIC (Gallic acid)
7.19e6
Gallic acid
2: MRM of 2 Channels ES+
TIC (5-HMF)
5.63e7
2.21
5-(Hydroxymethyl)furfural
20: MRM of 5 Channels ES-
TIC (Geniposidic acid)
1.60e6
2.49
Geniposidic acid
24: MRM of 5 Channels ES-
TIC (Oxypaeoniflorin)
2.29e6
3.54
Oxypaeoniflorin
7: MRM of 3 Channels ES+
TIC (Loganin)
2.23e6
4.17
Loganin
21: MRM of 6 Channels ES-
TIC (Geniposide)
5.89e5
4.20
Geniposide
23: MRM of 3 Channels ES-
TIC (Paeoniflorin)
2.13e6
4.75
Paeoniflorin
11: MRM of 5 Channels ES+
TIC (Ecdysterone)
1.07e7
5.25
Ecdysterone
17: MRM of 5 Channels ES+
TIC (Verbascoside)
1.55e6
5.32
Verbascoside
15: MRM of 5 Channels ES+
TIC (Cornuside)
2.69e6
5.92
Cornuside
25: MRM of 4 Channels ES-
TIC (Benzoylpaeoniflorin)
5.20e5
7.98
Benzoylpaeoniflorin
4: MRM of 3 Channels ES+
TIC (Paeonol)
7.85e7
8.98
Paeonol
13: MRM of 5 Channels ES+
TIC (Alisol B Acetate)
2.32e5
Alisol B acetate
100
%
0
Time
1.00 2.00 3.00 4.00 5.00 6.00 7.00 8.00 9.00 10.00 11.00 12.00 13.00 14.00 15.00 16.00

(B)

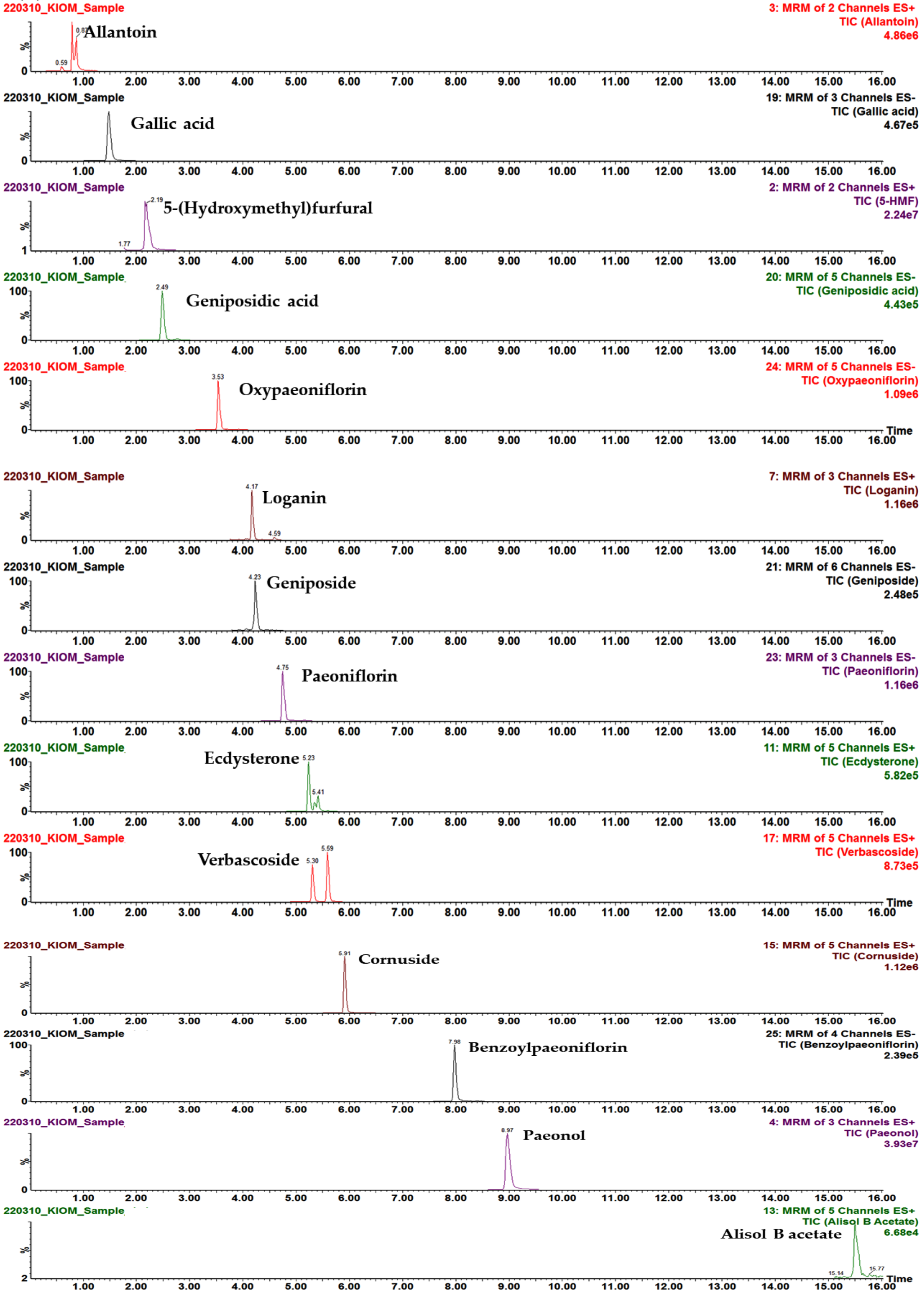
220310_KIOM_Sample
3: MRM of 2 Channels ES+
TIC (Allantoin)
4.86e6
Allantoin
220310_KIOM_Sample
19: MRM of 3 Channels ES-
TIC (Gallic acid)
4.67e5
Gallic acid
220310_KIOM_Sample
2: MRM of 2 Channels ES+
TIC (5-HMF)
2.24e7
5-(Hydroxymethyl)furfural
220310_KIOM_Sample
20: MRM of 5 Channels ES-
TIC (Geniposidic acid)
4.43e5
Geniposidic acid
220310_KIOM_Sample
24: MRM of 5 Channels ES-
TIC (Oxypaeoniflorin)
1.09e6
Oxypaeoniflorin
220310_KIOM_Sample
7: MRM of 3 Channels ES+
TIC (Loganin)
1.16e6
Loganin
220310_KIOM_Sample
21: MRM of 6 Channels ES-
TIC (Geniposide)
2.48e5
Geniposide
220310_KIOM_Sample
23: MRM of 3 Channels ES-
TIC (Paeoniflorin)
1.16e6
Paeoniflorin
220310_KIOM_Sample
11: MRM of 5 Channels ES+
TIC (Ecdysterone)
5.82e5
Ecdysterone
220310_KIOM_Sample
17: MRM of 5 Channels ES+
TIC (Verbascoside)
8.73e5
Verbascoside
220310_KIOM_Sample
15: MRM of 5 Channels ES+
TIC (Cornuside)
1.12e6
Cornuside
220310_KIOM_Sample
25: MRM of 4 Channels ES-
TIC (Benzoylpaeoniflorin)
2.39e5
Benzoylpaeoniflorin
220310_KIOM_Sample
4: MRM of 3 Channels ES+
TIC (Paeonol)
3.93e7
Paeonol
220310_KIOM_Sample
13: MRM of 5 Channels ES+
TIC (Alisol B Acetate)
6.68e4
Alisol B acetate
Time
1.00 2.00 3.00 4.00 5.00 6.00 7.00 8.00 9.00 10.00 11.00 12.00 13.00 14.00 15.00 16.00

Figure S6. Extracted ion chromatograms of each marker compound (A) and GSH sample (B) using the UPLC–MS/MS MRM method in the positive and negative ion modes.

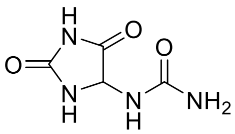

Allantoin (**1**)

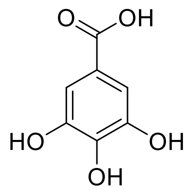

Gallic acid (**2**)

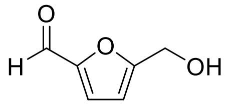

5-(Hydroxymethyl)furfural (**3**)

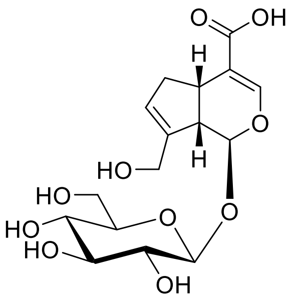

Geniposidic acid (**4**)

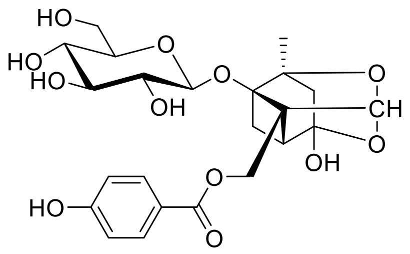

Oxypaeoniflorin (**5**)

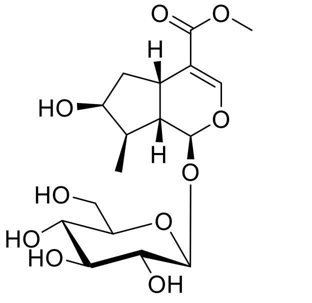

Loganin (**6**)

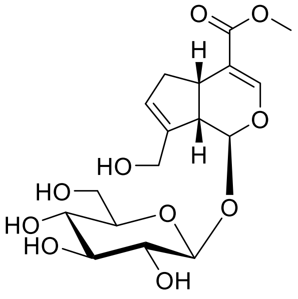

Geniposide (**7**)

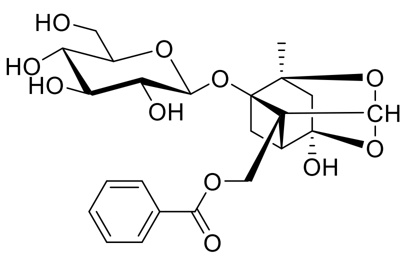

Paeoniflorin (**8**)

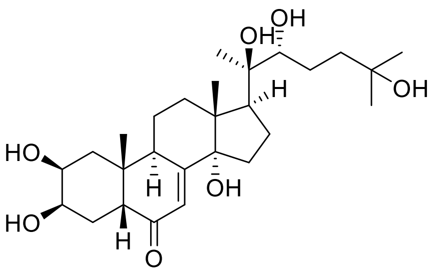

Ecdysterone (**9**)

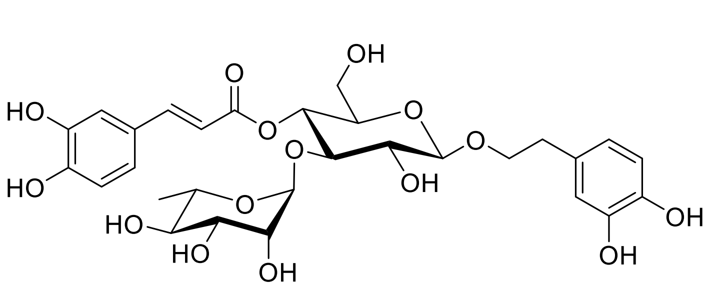

Verbascoside (**10**)

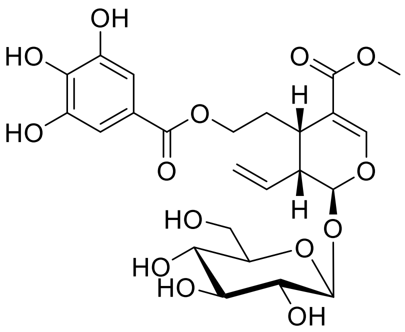

Cornuside (**11**)

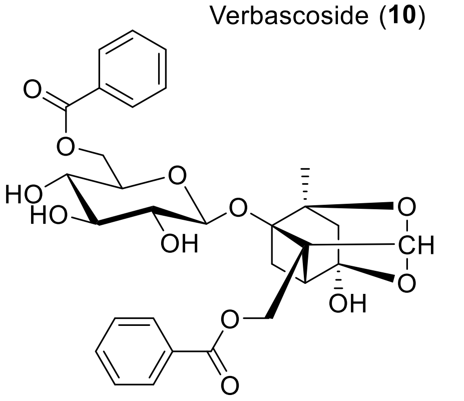

Benzoylpaeoniflorin (**12**)

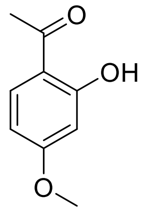

Paeonol (**13**)

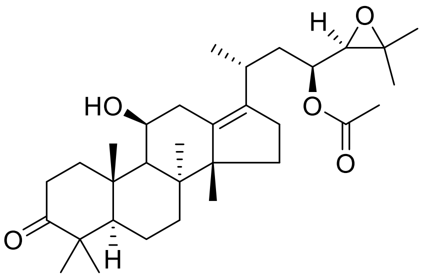

Alisol B acetate (**14**)

Figure S7. Chemical structures of the 14 marker components in GSH.
